# Formulations of Rancid and Winey-Vinegary Artificial Olfactory Reference Materials (AORMs) for Virgin Olive Oil Sensory Evaluation

**DOI:** 10.3390/foods9121870

**Published:** 2020-12-15

**Authors:** Ramón Aparicio-Ruiz, Sara Barbieri, Tullia Gallina Toschi, Diego L. García-González

**Affiliations:** 1 Instituto de la Grasa (CSIC), Campus Universidad Pablo de Olavide—Edificio 46, Ctra. de Utrera, km. 1, 41013 Sevilla, Spain; dlgarcia@ig.csic.es; 2 Department of Analytical Chemistry, Universidad de Sevilla, Prof. García González 2, 41012 Sevilla, Spain; 3 Department of Pharmacy and Biotechnology, Alma Mater Studiorum—Università di Bologna, Via della Beverara 123, 40131 Bologna, Italy; 4 Department of Agricultural and Food Sciences, Alma Mater Studiorum—Università di Bologna, Viale Fanin 40, 40127 Bologna, Italy; tullia.gallinatoschi@unibo.it

**Keywords:** virgin olive oil, reference materials, winey-vinegary defect, rancid defect, sensory assessment, volatile compounds

## Abstract

Sensory assessment of virgin olive oil (“panel test”) is the only sensory method included in international regulations of edible oils and its application is compulsory. Even if its application has been a success in quality control, improving the quality of virgin olive oils over the last 30 years, at present, there is no reference material (RM), in the strict sense of the term, to be used as a validated standard for sensory defects of virgin olive oil with which tasters can be trained. Usually, real samples of virgin olive oils assessed by many panels for the International Olive Council (IOC) ring tests are used as materials of reference in panel training and control. The latter are highly representative of the main perceived defects, but availability is limited, samples are not homogeneous year after year, and other secondary defects can be present. Thus, in order to provide solutions, this work describes an analytical procedure for implementing olfactory formulations that emulate rancid and winey-vinegary defects found in virgin olive oils with the aim of providing reproducible RMs that can be prepared on demand. A strategy for designing RMs for aroma is presented and the optimization process to obtain the best formulation is described. Under the criteria of representativeness, verified with the advice of the IOC, aroma persistence, and simplicity in formulation, two RMs for winey-vinegary and rancid were obtained by diluting acetic acid and ethanol (winey-vinegary defect) and hexanal (rancid defect) together with other compounds that are used to modify aroma and avoid non-natural sensory notes.

## 1. Introduction

A reference material (RM) is a requirement for different kinds of measurements because it allows different methods to be established for quantitation adjustment, reproducibility evaluation, and assessment of adequacy of the method for the analyte measurements [1,2]. Today, there is still a need to develop RMs in many applications. The procedure and definitions are clear since there are some regulations [3,4]. However, there are many difficulties in developing RMs in some cases due to variability of the matrix, its stability and interaction with compounds, and difficulty in synthesizing compounds with adequate purity, etc. A RM is defined as a material, sufficiently homogeneous and stable with respect to one or more specified properties, which has been established to be fit for its intended use in a measurement process [5]. There are two categories, RMs and certified reference materials (CRMs), which differ in the certificate that establishes the traceability to accurate realization and for which the certificate values are accompanied by an uncertainty at a defined level of confidence [3]. Thus, the process for the certification of a RM/CRM is well-established by a process that ensures traceability to accurate realization of the unit in which the property values are neatly expressed. RMs can be produced by institutions that are competent according to ISO 34 [4].

There are methods where the implementation of RMs are underdeveloped; for instance, methods on sensory assessment rarely use RMs in a strict sense according to the ISO definition [3]. However, sensory assessment, like any other metrology, requires that RMs provide qualitative and quantitative results. Furthermore, methods on sensory assessment of foods are not always included in trade standards and regulations or they are not described in detail. In the case of virgin olive oil (VOO), sensory assessment is a standard method, and its application is mandatory in business activities according to International Olive Council (IOC) norms and other national and international regulations [6,7].

In fact, there is a profuse legislation about the authenticity and quality characteristics of VOO categories [6] that is accompanied by series of analytical methodologies to determine the values of the parameters characterizing this edible oil. The laboratories that implement the analytical methods must demonstrate their proficiency in applying testing methods, following the procedures described by EU [8] and IOC [9], with trials that ensure the validity of the results. That validation process should be carried out with RMs, ideally CRMs, but there are not any CRMs to be used in the evaluation of sensory tasting laboratories or for training tasters.

Even if there is a good agreement among international regulations regarding the organoleptic quality of VOO [6,10] and the protocol for training tasters and sensory tasting laboratories [11,12,13], there are no RMs for sensory defects that are commonly detected in non-extra-VOOs (oils from virgin olive oil and lampante olive oil categories) to be used in the sensory analytical test for panel training and control. Instead, some natural VOOs are regularly selected as being representative of each sensory defect. They are considered as reference oils although their availability is limited, they are not homogeneous year after year, and other secondary defects can be present as well.

In 1991, the IOC method was included in European regulations and obtained legal validity to establish the quality grade of the product that included only three categories of VOOs: extra-virgin (EV), virgin (V), and lampante (L). Thus, in order to classify VOOs, a sensory profile sheet reports different oriented segments for assessment of intensity of the sensory descriptors in VOOs. The values, expressed as centimeters, are statistically processed to calculate the median of each positive (fruity, bitter and pungent) and negative attribute (main defects: fusty-muddy, winey-vinegary, rancid, musty, frostbitten olives) [11,12].

The current legislation [8] states that the median of the defects has to be zero in EVs or, in other words, that no intensity of any sensory defect should be detected. In addition, the median of the fruity attribute also has to be higher than zero in EVs. EU Regulations also state that the other categories of VOOs (V and L) are qualified with a median of the defects that varies from very slight for V (Median ≤ 3.5) to high enough for L (Median > 3.5) (EEC Regulation 2568/1991 [14] and subsequent amendments).

Some difficulties persist, however, in perceiving and distinguishing a specific defect in VOOs when the defect intensity is very slight (e.g., close to 0.0 median). A debated classification can also occur in the case of oils that have an intensity of the defect close to the limit between VOO and LOO [15,16,17].

Additionally, the difficulty in recognizing a sensory defect at low intensity with respect to other sensory characteristics in the oil is also given and may add more uncertainty to the evaluation. This is particularly true in the case of oils that have more than one defect and for which the classification depends on the agreement reached by the tasters not only on the intensity, but also on the type of the defect perceived [15].

In this regard, in a recent work [16] on the alignment and the proficiency of VOOs sensory panels, the authors proposed several tools for quality control (i.e., a new decision tree scheme and formative reassessments) that allow a reliable classification of samples and highlighted the need for new reliable reference materials to enhance the skills of the panel in recognizing, identifying, and quantifying sensory attributes, a statement with which other authors have agreed [15]. Currently, in order to control these aspects, to train panelists and to harmonize odor thresholds, reference oils are regularly provided by the IOC or are obtained by the panel leader and are considered as RM. These RMs are VOOs that are selected as they possess a sensory defect that is clearly perceived and is not mixed with other sensory defects according to the assessment of accredited sensory testing laboratories. These characteristics are often difficult to find and the materials found every year for a single sensory defect are characterized by different sensory notes, which is against of the definition of a RM that should be the same every year. Thus, this fact prevents comparability of the results between years and tracking of the panelist responses over time.

The high variability of RMs and the impossibility for all panels to use the same material over the years make harmonization of panels difficult to accomplish. Thus, RMs in any analytical method, if they are homogeneous and sufficiently stable, would allow for the determination of cut-off thresholds and aid in data handling and interpretation of results. This work describes the full process for elaboration of two formulations to be used as RMs for sensory training for winey-vinegary and rancid aromas in VOOs. Because good reproducibility over the years is needed, the production of RMs was based on volatile compounds instead of natural VOOs to avoid variability between batches. Thus, RMs emulating the aromas of rancid and winey-vinegar defects were produced using a collection of synthetic volatile compounds diluted at specific concentrations. The process needed to define a strategy to formulate RMs that emulate aroma. Considering that there is scarce experience on formulating such a RM and that RMs for sensory assessment are underdeveloped with respect to other analytics, a flow diagram is necessary considering the large knowledge on the volatile composition of VOOs [18,19,20,21,22]. The starting point was the experience of the authors in selecting volatile markers for sensory defects [18,23], including the verification of their sensory profile by gas chromatography (GC)-olfactometry and other sensory studies [18], as well as their quantification by analytical procedures [24,25]. Although key odorants are always present when a sensory defect is perceived, they do not necessarily smell like the sensory defect. In fact, a sensory defect may be caused by marker compounds in combination with others in a synergetic process. Thus, in sensory terms, reproducing the aroma of a sensory defect is not as easy as mixing these markers or key odorants in refined oil. On the contrary, it requires studying the interaction aroma-aroma phenomena. The strategy to reproduce aroma by flavor matching, through solving problems and facing challenges, is described herein.

## 2. Materials and Methods

### 2.1. Reagents

The following compounds (food grade, purity in parenthesis) were purchased from Sigma-Aldrich (St. Louis, MO, USA): ethyl acetate (≥99%), ethanol (96%), hexanal (≥97%), 4-methylpentan-2-ol (98%), *E*-2-hexenal (≥95%), hexan-1-ol (≥98%), *E*-3-hexen-1-ol (97%), *E*,*E*-2,4-hexadienal (95%), nonanal (≥98%), acetic acid (≥99.5%), *E*-2-decenal, pentanoic acid (≥99%), hexanoic acid (≥98%), and *E*,*E*-2,4-decadienal (≥89%).

### 2.2. Samples

Full refined olive oils (flavorless) were obtained from Aceites del Sur (Sevilla, Spain). Oils were checked for small or null presence of volatiles by solid phase microextraction-gas chromatography-mass spectrometry (SPME-GC-MS) as well as for the absence of aroma (mainly rancidity) prior to be used as a matrix to formulate RMs. Sixty-three samples of VOOs (12 EV, 32 V, 19 L) were collected from olive oil producers for analysis by solid phase microextraction-gas chromatography-flame ionization detector (SPME-GC-FID) and to check for the presence of the most relevant volatile markers. The 51 samples of V-L included 20 and 7 samples with rancid and winey-vinegary, respectively, as the main perceived defect. The remainder of the V-L samples presented the following defects (number of samples between parenthesis): fusty-muddy (18), musty-humid-earthy (3), frostbitten olives (1), and brine (2). Two VOOs used as references in panel training and testing were supplied by the IOC and were also used as guiding samples to prepare tentative mixtures of volatiles emulating sensory defects. These samples were a rancid standard qualified with a median of defect (Md) of 9.5 and a standard of winey-vinegary with a Md of 5.7.

All the samples were kept at −20 °C before use in experiments.

### 2.3. Determination of Volatile Compounds

The procedure for determination of volatiles [26,27] consisted of two steps: concentration of volatiles and their quantification by gas chromatography. The concentration step was carried out on a MultiPurpose Sampler (Gerstel GmbH, Mülheim an der Ruhr, Germany). Samples (2 g spiked with 2.6 mg/kg 4-methyl-2-pentanol as internal standard) were placed in a 20 mL glass vial, tightly capped with polytetrafluoroethylene (PTFE) septum, and left for 10 min at 40 °C to allow for equilibration of volatiles in the headspace. After the equilibration time, the septum covering each vial was pierced with an SPME needle, and the fiber was exposed to the headspace for 40 min. The SPME fiber (1 cm length and 50/30 μm film thickness) was purchased from Supelco (Bellefonte, PA, USA) and was endowed with the Stable Flex stationary phase of divinylbenzene/carboxen/polydimethylsiloxane (DVB/CAR/PDMS). The fiber was conditioned following the instructions of the supplier.

Once the concentration step was terminated [28], the volatiles absorbed by the fiber were thermally desorbed in the hot injection port of a GC for 5 min at 260 °C with the purge valve off (splitless mode) and deposited onto an Agilent J&W DB-WAX column (60 m; I.D. 0.25 mm; film thickness 0.25 µm; Agilent, Santa Clara, CA, USA) of a GC-MS/FID (7820A Agilent Technologies gas chromatography coupled to a Series MSD 5975 Agilent Technologies mass spectrometry). The carrier gas was hydrogen, at a flow rate of 1.5 mL/min. The oven temperature was held at 40 °C for 10 min and then programmed to increase by 3 °C/min to a final temperature of 200 °C. The signal was recorded and processed with MSD ChemStation E.02.02.1431 (Agilent Technologies Inc., Santa Clara, CA, USA. The results were expressed as relative areas with respect an internal standard.

### 2.4. Sensory Analysis and Optimization of RM Formulations

Nine trained assessors (panelists) smelled the formulations of RMs (5 g) from opaque vials (25 mL). The vials were closed for >40 min prior to being tested. The sensory evaluation by the panelists focused on the adequacy of the RM compared with a VOO sample presenting the sensory defect (winey-vinegary or rancid) that was also smelled together with the formulations. The presentation of RMs to assessors was carried out for 2–4 times depending on the homogeneity of the responses. All samples were presented blinded and the assessors did not know the specific defect that was being emulated. After sensory evaluation of the formulations, discussion ensued and the information extracted was considered to make decisions on compositional changes.

To establish an order in the compositional changes, an experimental design based on modified simplex was carried. Modified simplex [29] is an experimental design procedure that allows easy implementation and wide choice of methods for optimization. Three characteristics, called “desirabilities”, were selected as variables to be optimized: odor representativeness, odor persistence, and formulation simplicity, with the first two being evaluated by assessors. They can be described as follows: a chemical formulation should have an odor as similar as possible to the sensory defect perceived in real VOOs, the odor persistence of the formulation should be as long as possible and at enough intensity, and the formulation should have a composition with the maximum simplicity possible (less than 10 compounds to allow easy and reproducible preparation of the RM). Within the first desirable properties (representativeness), the absence of chemical aroma or detection of being non-natural were also evaluated. The decision steps were focused on qualitative “yes/no” criteria with specific questions: “Is the aroma representative?”, “Is the aroma natural?”, “Is its persistence enough?”, “Is the formulation simple?”. Depending on the compliance of these “desirabilities”, the concentration of the compounds was decreased or increased and at certain points, and the sensory description reported by assessors determined the inclusion/exclusion of volatile compounds.

## 3. Results and Discussion

### 3.1. Planning and Selection of Volatile Markers Suitable for Formulations

The objective of preparing mixtures of chemical compounds to provide aromas that resemble sensory defects in VOOs can be carried out using volatile compounds that do not necessarily have to be among those identified in VOO or by using a matrix as carrier that can be different from the refined olive oil [15]. However, in order to obtain greater representativeness of the aroma, the objective of this work was addressed using volatiles identified in VOOs and were preferentially markers of the two sensory defects considered, i.e., rancid and winey-vinegary [18]. It is important to remember that a marker of a particular sensory defect is a compound that is identified in samples with that sensory defect and its presence is related to the associated off-flavor in an almost indubitable manner. When the sensory defect is perceived by a panelist when smelling a VOO, this marker should be identified. There is, in consequence, a causal relationship between the identification of this compound and the sensory defect or the biochemical/chemical processes that give rise to it [30,31]. However, it does not mean that this compound is necessarily the only one responsible for the complex sensory notes defining the defect. In fact, the aroma perceived by the compound can be masked by other chemical compounds that also contribute to the aroma. Furthermore, in some cases, the aroma provided by the volatile marker can be far from the aroma of the corresponding sensory defect. In that case, the marker is useful to identify the presence of a sensory defect, because this compound is associated with the chemical/biochemical process causing the sensory defect, but it would not be suitable to emulate the sensory defect aroma (henceforth target aroma). Thus, under this premise, the RM can also be prepared with volatile compounds that may not be the most informative markers of the sensory defect but they can contribute in reproducing or enhancing the sensory descriptor of the target aroma.

**Figure 1:**
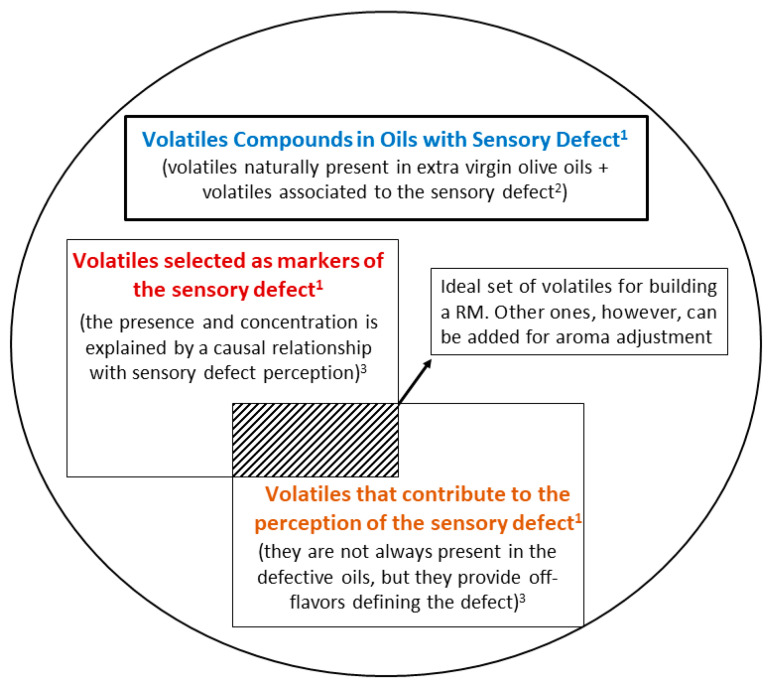
Scheme to select volatile compounds as candidates to formulate RMs of aroma sensory defects detected in VOOs. Note: ^1^ in this work sensory detect refers to rancid or winey-vinegary; ^2^ volatiles can result from oxidation (rancid defect) or fermentation (winey-vinegary defect) processes; ^3^ the concentration of these compounds may exceed or not their odor threshold, and can or cannot smell like the sensory defect.

Figure 1 shows the scheme of the strategy used. The volatile compounds that are suitable to formulate the RMs are those resulting from the overlap between two subgroups of volatile compounds: sensory markers of the defect (subgroup 1), though not all can contribute to the sensory perception, and those that can contribute to the aroma of the sensory defect (subgroup 2), even if not all contribute exclusively to it or are always identified in oils with the corresponding sensory defect. In other words, the compounds that lie in the overlap of these two subgroups are those that provide aromas that are highly representative of the off-flavor of the sensory defect, regardless whether they are always present in natural VOOs with the defect.

In addition, the strategy included the possibility to add other compounds that are not related with the defect, but that can be used to modulate the perception of certain sensory notes. These compounds can also be used to avoid “a chemical perception” and give a more “natural” aroma to the RM considering all the nuances of the target aroma.

**Figure 2:**
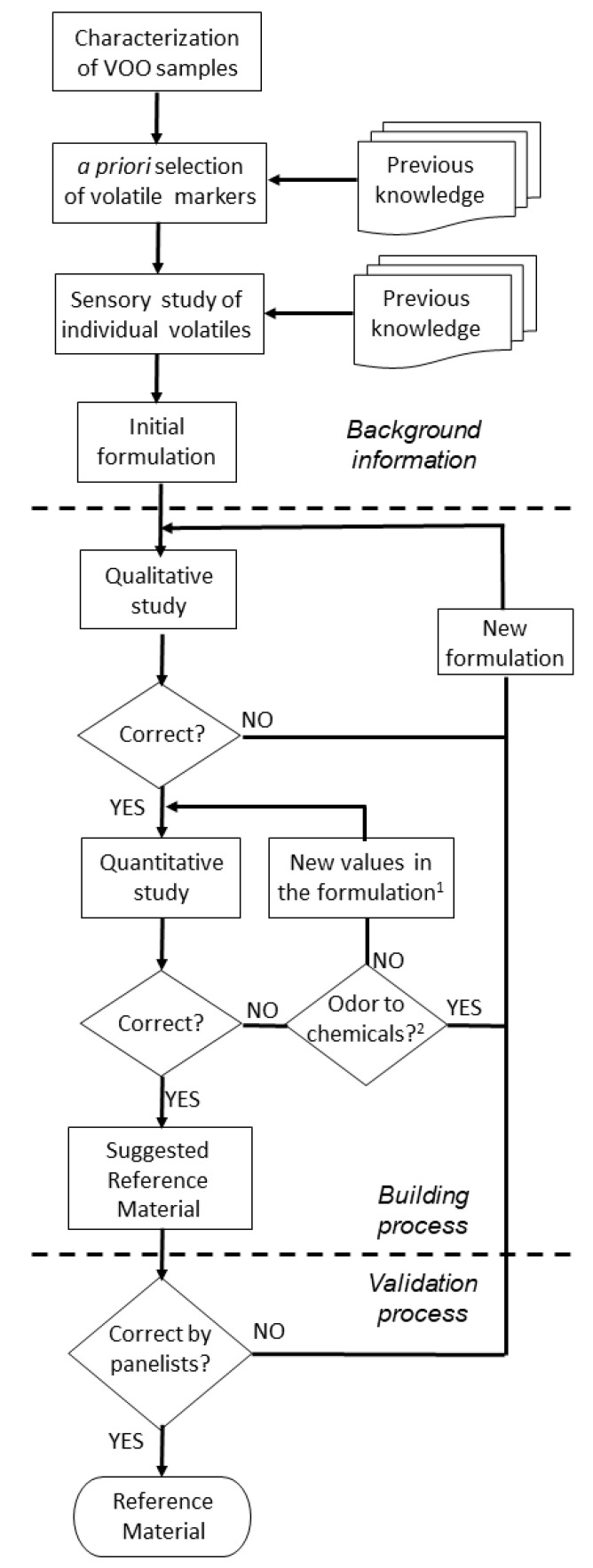
Flow diagram for formulating RMs. Note: ^1^ reformulation includes selection/removal of compounds and/or new adjustment of concentrations; ^2^ the question refers to perception of a “chemical” aroma that is far from the natural off-flavors found in VOO.

The process to produce optimal formulations of RMs for rancid and winey-vinegary defects required a flow diagram to make use of previous knowledge on the relationship between volatile compounds and sensory descriptors of VOOs [18]. Figure 2 shows the flow diagram designed for the development of RMs for sensory defects of VOOs. The background information refers to previous studies that established the scientific basis for the identification of volatile compounds that are responsible for several sensory defects and the determination of their concentration thresholds that contribute to the aroma of those VOO defects [18,22,32,33].

Table 1 shows the main volatile markers selected for the two sensory defects studied, winey-vinegary and rancid. In the case of winey-vinegary defect, the individual dilutions (1000 mg/kg) of compounds determined that the aroma provided by acetic acid, ethyl acetate, and ethanol were highly related with the aroma of the sensory defect when this dilution was evaluated by panelists with a scale of three degrees (low-medium-high representativeness). Consequently, they were selected to begin the formulations and were nominated as “primary compounds” whose aroma could be modified by the addition of others. In particular, acetic acid and ethyl acetate were related with the winey aroma, while ethanol was more related to fermentative and winey notes. The compound 3-methyl-1-butanol was excluded since its aroma was considered far from the target aroma.

In the case of rancid defect, all the compounds were characterized by a high representativeness and all provided similar aromas (oily, fatty), except for *E*-2-decenal (fishy, fatty) and hexanoic acid (rancid). Hexanal and nonanal were selected as “primary compounds” to prepare the first formulations because of their demonstrated relationship with lipid oxidation and the rancidity process of fatty foods [34,35], including VOO [18,36,37,38].

The aforementioned volatiles were considered as primary compounds and were identified in 63 VOOs that covered the three categories (EV, V, L) and all possible qualities. Table 2 shows the relative areas of these compounds. The study was based on relative areas instead of quantification by calibration curves, since the range of possible concentration values reached high values (in some cases with median of the defect around 8) and therefore competition phenomena may alter the accuracy of the quantification. The objective was to establish the high range of relative areas that needs to be reproduced in a formulated RM using the same volatile compounds at similar or higher concentration. Thus, the relative areas for acetic acid were higher in V and L oils characterized with winey-vinegary defect (median of the defect = 1.7–3.8). Nevertheless, higher relative standard deviation (8.485) was found in a sample without this sensory defect, which shows the importance of the masking effect of other compounds when defining the sensory profiles of VOOs. The relative area of ethanol was also higher in oils with winey-vinegary defect. However, this compound is naturally present in VOO and is additionally produced by fermentation. This explains why the maximum content (relative area 1119.060) was found in an oil without this sensory defect (its defect was fusty-muddy). This compound was considered as an outlier to calculate the median in Table 2, although the high content of ethanol can be easily found in VOOs with fermentative defects. The content of ethyl acetate was also higher in VOOs with winey-vinegary defect. In fact, the maximum content (relative area 3.149) was found in a sample with this sensory defect. The relative areas of these compounds in a RM of winey-vinegary defect provided by the IOC revealed a high content of acetic acid and ethyl acetate (even four and six times, respectively, the maximum content found in the 63 VOOs). On the contrary, a low content of ethanol was found, even lower than some relative areas found in EVs.

Regarding the two volatile compounds selected for rancid defect, hexanal showed a higher mean of relative area in rancid oils. However, the maximum content was found in VOOs with no rancidity and the minimum content was found in VOOs with rancid defect. This observation is explained by the dual contribution of this compound to green fruitiness aroma, on one hand, originating from the lipoxygenase pathway, and rancidity, on the other hand, when it reaches a high concentration as a consequence of lipid oxidation [39]. Nonanal showed the highest relative areas in VOOs with rancidity, and its maximum in these oils was 10 times higher than the maximum in the VOOs without this sensory defect. The RM of rancid oil provided by the IOC showed relative areas of hexanal and nonanal near the maximum found in VOOs (1.33 and 1.45 times the relative area of the maximum found in the 63 VOOs, respectively).

The flow diagram shown in Figure 2 starts with the preparation of a first formulation with the selected volatile markers (Table 2) to emulate a particular sensory defect (rancid or vinegary) with the aim of carrying out an iterative process to optimize composition and concentrations. This phase, described in the following two sections for winey-vinegary and rancid defects, was called the “building process” (Figure 2). In this phase, the first formulation was submitted to the experimental design of modified simplex [29]. In each iterative step of the modified simplex, a set of trained assessors determined three described desirability properties (see Section 2.4): representativeness, odor persistence, and formulation simplicity. The modification of the formulation was based on both qualitative (inclusion/exclusion of volatile compounds) and quantitative information (concentration of compounds) until reaching agreement among trained panelists in the compliance of the formulation with the required “desirabilities” of representativeness, odor persistence and simplicity (see Section 2.4). The modified simplex procedure allows adjustment of the concentration in a systematic manner and to understand how the concentrations of volatiles needed to be changed.

With this procedure, new optimized formulations from each set of experiments were produced, and, finally, the two selected formulations of each set of experiments, with the highest possibilities of being a RM, were sensory evaluated by a set of Italian and Spanish assessors.

The last phase of the flow diagram (Figure 2) involves evaluation of the best RMs by panel tests and optimization of their use in training and panel monitoring. This step is not described herein, although it is being carried out within the OLEUM project (Horizon2020, Gran Agreement No. 635690).

### 3.2. Building a Formulation as RM for the Winey-Vinegary Defect

**Figure 3:**
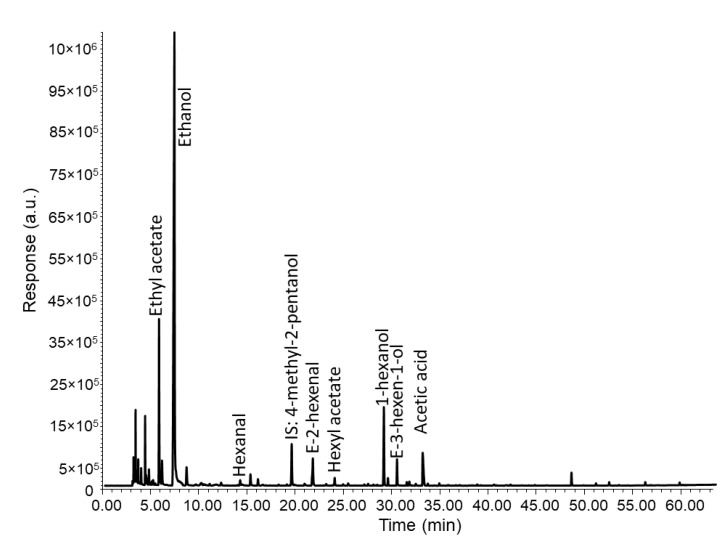
Chromatogram of a VOO qualified with an intensity (median of the defect) of 5.9 of the winey-vinegary aroma defect.

As a first step, the chromatograms of oils with winey-vinegary defect were examined to identify the most relevant volatile compounds. Figure 3 shows the chromatogram of a VOO that was qualified with a medium-high intensity (Md = 5.7) of vinegary-winey defect by sensory laboratories. There were 7 major and most characteristic volatiles, although only three (ethyl acetate, ethanol and acetic acid) explain the vinegary-winey defect. These volatiles were selected as “primary compounds” for this sensory defect since they smell sticky, alcohol/spicy/winey, and vinegary, respectively [40]. The other four (hexanal, *E*-2-hexenal, 1-hexanol, *E*-3-hexen-1-ol) contribute to cut grass/green sensory perception, a perception that is present in all VOOs at different levels of intensity.

The first conclusion is that none of the three “primary volatiles” selected for vinegary aroma in VOOs do not necessarily smell like the sensory defect by itself, although their aroma partially resembles sensory notes perceived in winey-vinegary oils. They are considered volatile markers because they are always present when the aroma is perceived (Table 2), and furthermore there is a causal relationship between the intensity of the vinegary aroma and the concentration of the marker. Thus, the final aroma of a VOO qualified with the winey-vinegary defect results from the presence of its volatile markers in combination with others. Thus, reproducing the aroma of the winey-vinegary aroma in VOOs is not as easy as adding volatiles to a refined olive oil. On the contrary, it requires studying the aroma-aroma interaction in the process of flavor matching to reproduce the aroma of a RM. Thus, when mixing volatile compounds to formulate a RM, the resulting aroma should be perceived with a holistic perception (a single aroma), just as it occurs in a real VOO, instead of an analytical perception (several aromas mixed that can be identified in the mixture).

To create the first formulation, the seven volatile compounds were first diluted to 300 mg/kg and the concentrations were reduced/increased to reach the intensity levels found in a real VOO. The final concentrations were as follows: ethyl acetate (80 mg/kg), ethanol (600 mg/kg), acetic acid (40 mg/kg), 1-hexanol (20 mg/kg), and *E*-3-hexen-1-ol (40 mg/kg). Hexanal and *E*-2-hexenal were initially excluded, since they gave rise to an aroma similar to 1-hexanol and *E*-3-hexen-1-ol and would be included in further steps if the optimization of the formulation required adding new compounds to provide green aroma.

The mixture of these compounds provided the first formulation, coded as AV1 (Table 3). The first conclusion was that the aroma as perceived by assessors was not homogeneous. The initial formulation AV1 was characterized by a slightly vinegary aroma, although the assessors reported sweet sensory notes (two assessors) and a slight rancid odor (three assessors).

After obtaining individual opinions from assessors, it was decided to modify the composition before submitting the formulation to the modified simplex. In particular, the concentrations of the volatiles responsible for sensory defects (ethyl acetate, ethanol, acetic acid) was increased with the aim of producing an aroma that all the assessors would relate with winey-vinegary defect. Thus, acetic acid and ethanol were diluted to 600 mg/kg and ethyl acetate to 200 mg/kg. However, the assessors unanimously qualified this aroma as “chemical” and “non-natural”, and it was therefore decided to increase the concentrations of the compounds producing green/fruity aroma (hexanal, *E*-2-hexenal, 1-hexanol, *E*-3-hexen-1-ol) to obtain a “green base aroma” that reminds of VOO.

In order to optimize the concentrations of the volatile compounds that are responsible for fruity perceptions (green, fruity) to build a proper “green base aroma”, the four compounds—1-hexanol, *E*-3-hexen-1-ol, hexanal, *E*-2-hexenal—were diluted together at two different concentrations for each compound: 50 mg/kg and 35 mg/kg. The formulation at 50 mg/kg was qualified by all assessors as “a neat green with a bit almond”. However, two assessors reported “rancid” sensory notes that are characteristic of hexanal and *E*-2-hexenal at high concentrations. However, a decrease of 30% in the concentration of these volatiles (35 mg/kg) widely modified the sensory perception of the formulation by assessors who qualified it as “green with a bit almond perception”. The assessors reported that they recognized the characteristic aroma of *E*-2-hexenal (green almond), which seems to have be prevalent over the other aromas (more green or green apple). No rancid notes were detected. However, in terms of representativeness, the aroma of the formulation at 50 mg/kg was considered more natural and closer to VOO than the formulation at 35 mg/kg. Consequently, the concentration of 50 mg/kg was selected as the starting concentration for providing the “green base aroma”.

A mixture of the “green base aroma” together with the three primary compounds at high concentration (acetic acid and ethanol diluted at 600 mg/kg and ethyl acetate at 200 mg/kg) was prepared, coded as AV2 (Table 3), and submitted to the modified simplex procedure [41]. The modified simplex produced a series of formulations (Table 3) with concentrations of the “primary volatile compounds” that were responsible for detection in the high range (>600 mg/kg) mixed with several levels of concentrations of the four volatiles responsible for the green perception at the low concentration range (<50 mg/kg). The formulations coded as AV2-AV5 were rejected by assessors as having low representativeness of the aroma presenting sensory notes that were not related with winey-vinegary defect. Moreover, some glue, rancid, and wood notes were detected. Furthermore, the winey-vinegary notes were detected at low intensity and this aroma was identified as “non-natural”. The required characteristic of odor persistence was not satisfied due to the low intensity of the aroma.

These four formulations were composed of diverse concentrations of the three primary volatile compounds: AV2 and AV4 were prepared with ethyl acetate at medium concentration (200 mg/kg) and ethanol and acetic acid at low concentration (600 mg/kg). The formulation AV3 was made with higher concentrations of ethyl acetate (400 mg/kg) and ethanol and acetic acid (1200 mg/kg). Finally, the formulation AV5 was prepared with much lower concentration of ethyl acetate (40 mg/kg), which was identified as a compound that produced a non-natural aroma. In this formulation, ethanol was added at very high concentration (5000 mg/kg) since this compound was identified as an enhancer of the vinegary perception produced by acetic acid, whose concentration was kept low (600 mg/kg). The concentration of the set of volatiles responsible for green perception was also reduced to avoid the rancid perception reported by some assessors and to avoid masking the winey-vinegary defect, which was considered as non-evident by assessors in these formulations.

In the evaluation of these four formulations (and in others not shown in Table 3), some conclusions were extracted. First, the sensory evaluation of these formulations did not result in a unanimous evaluation and sensory notes that were not related with winey-vinegary defect (rancid, wood and mushrooms aromas) were identified by some assessors, while vinegary sensory note was only reported as “slight intensity” and “non-natural”. Secondly, all assessors agreed in the characteristics of “non-natural” and low representativeness of the aromas, and the sensory notes of glue and chemicals were identified as the reason for this non-natural aroma. Individual dilutions of compounds identified ethyl acetate as being responsible for these sensory notes, particularly at concentrations higher than 80 mg/kg. It was thus decided to remove this compound from subsequent formulations. The third conclusion was that the increase of the concentration of acetic acid did not lead to a proportional increase of the intensity of vinegary aroma. On the contrary, the mixture of acetic acid and ethanol at different concentrations led to abrupt changes of the perception of the vinegary aroma, which explained the increase of the concentration of ethanol as aroma enhancer. The fourth conclusion was that the volatiles responsible for green aroma at a concentration of >50 mg/kg, although they contribute to the natural aroma similar to VOO, they also have a negative effect on the aroma. This negative effect consists in producing an “analytical” perception of the aroma, in which the individual effect of some compounds is perceived with rancid notes. This effect was considered as undesirable since the goal was to produce an aroma characterized with a “holistic” perception (i.e., no individual contribution of the compounds diluted in the formulation) of a winey-vinegary aroma as it occurs in natural VOOs.

A programmed set of formulations based on modified simplex design using AV4 led to AV5 and AV6 (Table 3). In these two formulations, the addition of ethyl acetate was omitted because of its contribution to “non-natural” perception, even if it is an excellent marker of winey-vinegary defect and was initially identified as a “primary compound” to emulate the aroma of this defect. The new strategy for subsequent steps (AV7-AV9, Table 3) in modified simplex design was based on increasing the concentrations of all the volatiles as described in AV6.

Table 3 shows the full agreement of the assessors in evaluation of these three formulations. In these formulations, the assessors clearly identified vinegary aroma and did not detect any masking effect or interferences from other compounds. From these formulations, AV7 and AV8 were considered by all assessors as satisfactory in terms of representativeness and aroma persistence, keeping the composition simple as in the previous formulations. AV8 was characterized with artificial sensory notes, although to a lower degree than in the previous formulation. However, assessors reported that the vinegary aroma in this formulation was more evident than in AV7, and, in consequence, AV8 was considered as the best formulation to be proposed as a RM of winey-vinegary sensory descriptor. When the headspace of this formulation was analyzed by SPME-GC-FID the relative chromatographic area of acetic acid was 9.700, close to the value for the IOC RM (8.688, Table 2). However, in the case of ethanol, its relative chromatographic area (75.81) was much higher than the IOC RM (0.581), although closer to the maximum values found in the winey-vinegary oils (19.039) and in oils with other defects (53.688) in the data set studied (Table 2).

### 3.3. Building a Formulation as RM for the Rancid Defect

**Figure 4:**
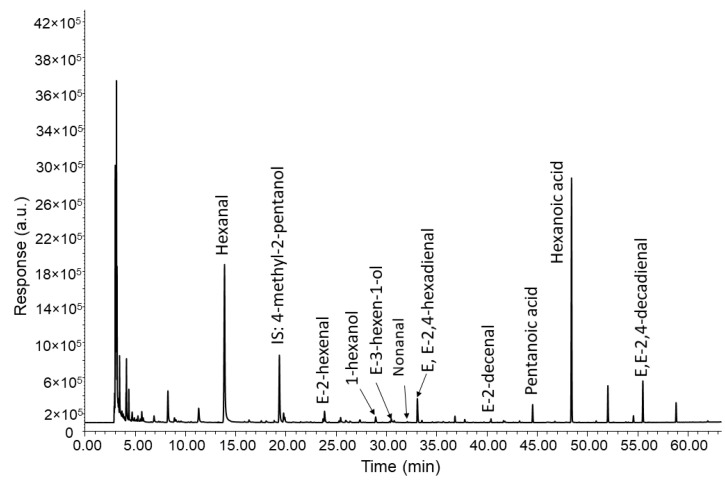
Chromatogram of a VOO qualified with an intensity (median of the defect) of 9.5 of the rancid aroma defect.

Figure 4 shows the chromatogram of a rancid VOO (Md = 9.5) in which relevant compounds were identified. To build a RM for this defect, the initial formulation (R1, Table 4) was prepared with the main markers of a rancid perception that were selected as “primary compounds” (hexanal, nonanal, Table 2) [18,22,37,38]. The dilution of these two compounds alone (600 mg/kg and 80 mg/kg for hexanal and nonanal respectively) produced a chemical perception far from actual VOOs, and was qualified as “chemical” or “non-natural” by assessors. Consequently, as in the formulations for winey-vinegary defect, this formulation also required addition of a “green base aroma”. In the initial formulation, *E*-2-hexenal was excluded from the compounds producing the green perception, since their aroma was distinctly perceived as green almond, and only two volatiles, 1-hexanol and *E*-3-hexen-1-ol, were added at 20 mg/kg and 40 mg/kg, respectively, to reproduce the green aroma. However, aldehydes, which are the main markers of the rancid perception because their concentrations mainly result from oxidation processes [39], are also associated with a green-fruity sensory note when they are present at low concentrations; hexanal is the paradigm. This simple fact means that if the concentration of aldehydes reaches the olfactory receptors of assessors at low concentrations, due to the dispersion of molecules in air, they will firstly perceive a green odor that can be followed by a rancid one once the concentration increases with time. The duality of aroma characteristics of hexanal under different concentrations and air flow rates was shown in a previous study in which assessors reported both rancid and green aroma [30]. Thus, R1 was qualified by most assessors as green aroma ending with a slight rancid perception (Table 4). Furthermore, it was observed that, at the same concentration, the prevalence of green or rancid perception greatly depended on the assessors, who repeatedly noted one of the two types of aroma when they smelled the formulation in repeated experiments. This observation pointed out a subjective dependence, which obliged us to reconsider the initial formulation to provide an aroma that the assessors defined as unquestionably rancid.

As a first approach for the reformulation, the combination of hexanal and nonanal to emulate rancid aroma was studied by sensory assessment of two dilutions at 600 mg/kg and 6000 mg/kg of both compounds individually. When hexanal was presented to assessors at 600 mg/kg, its aroma was perceived as spicy and green with a rancid perception at the end of the smelling. A sample of nonanal, at identical concentration, was qualified as rancid or highly rancid, wood, and green. The concentration of those volatiles at 6000 mg/kg was perceived as a highly rancid, flat odor followed by lard and tallow perceptions, but also green aroma was reported despite the high concentration. As a consequence, it was concluded that the aroma of these two compounds may confuse tasters when trained for the rancid descriptor. It was decided to remove nonanal from the formulation and keep hexanal, although the reverse alternative would also have been valid.

As a second approach to reformulate R1, a search for new compounds providing rancid aroma was carried out. Figure 4 shows the chromatogram of a VOO qualified with an intensity of rancid perception at Md = 9.5. The chromatogram shows that there are four other compounds (*E*,*E*-2,4-hexadienal, *E*,*E*-2,4-decadienal, *E*-2-decenal and hexanoic acid) that contribute to rancid perception without the problems of the aldehydes as already pointed out and led to a major consensus in sensory evaluation by assessors.

Thus, a new formulation was produced (R2, Table 4) in which *E*,*E*-2,4-hexadienal, *E*,*E*-2,4-decadienal and *E*-2-decenal was added at 600 mg/kg and hexanal was kept in the formulation although at low concentration (100 mg/kg). This new formulation was subjected to modified simplex. Concerning the set of volatiles to produce a green perception, the addition of these new aldehydes masked the bitter almond aroma of *E*-2-hexenal, and we opted for the pool of compounds used to formulate the vinegary defect. Formulations R2-R4 were sensory evaluated by assessors (Table 4) at three concentration levels (100, 50, and 35 mg/kg) of the compounds contributing to a green-fruity sensory perception (high –R2-, medium –R3- and low –R4), but without excessively masking the rancid perception, which was always perceived by all assessors. The sensory descriptions of the three formulations were similar, including green, rancid, and sweet notes (Table 4). However, when the concentrations of the compounds contributing to green aroma were reduced to 35 mg/kg, other sensory notes were perceived, such as ripe fruit, rancid nuts, and rancid aftertaste.

To emphasize the rancid perception over the green aroma, it was decided to change the formulation by adding other volatiles which are also responsible for the rancid defect. The compounds selected were pentanoic and hexanoic acids. Modified simplex allowed checking results of decreasing the concentration of volatiles contributing to green-fruity perception in order to avoid disagreement among assessors if there was or not masking of the rancid perception by the essential VOO green aroma. The assessors agreed that the best result in terms of representativeness and odor persistence was R6 (Table 2), which was considered as the most promising as a RM for rancid defect in VOOs. Regarding the “primary compound” in the formulation (hexanal), when this formulation was analyzed by SPME-GC–FID, the relative chromatographic area was 20.268, which was approximately twice the relative area found in the IOC RM (8.396), but lower than the maximum found in VOOs (164.316).

## 4. Conclusions

The formulation of mixtures to emulate aromas of foods requires combining knowledge from both compositional data (flavor compounds) and sensory analysis. Since the target was to reproduce the aroma of sensory defects at high intensity, the high concentrations of compounds renders volatile analysis difficult. However, the objective was not to reproduce the same volatile profile, but to obtain the same aroma with a reduced number of volatile compounds. Thus, the concentrations of the compounds were not limited by the natural values found in VOOs, but rather the concentrations needed to produce the aroma, overcoming problems of masking and enhancing the synergy. Working in the gap between chemical composition and aroma perception leads to some difficulties due to inherent particularities in the interface between chemical composition and physiological olfaction [30]. All of these aspects were carefully considered during formulation to avoid the risk of obtaining a non-representative RM. In summary, these particularities are: (1) the perception of the aroma of mixtures of volatile compounds, in particular when they are at high concentration, can be perceived as “non-natural” or “chemical” by assessors, even though the aroma of the mixture highly resembles the aroma of the sensory defect; (2) the perception of a mixture of volatile compounds can be “analytical” (different aromas are detected as parts of the global aroma) or “holistic” (a simple perception of one aroma resulting from the partial contribution of all compounds in the mixture), the second case being the desirable one; (3) the relationship of the response of human perception is non-linear with respect to increasing concentrations of substances; (4) the release of volatile compounds from an oily matrix has been scarcely studied (most studies were developed for water dilutions) and it concerns the temporal dimension of flavor perception; (5) some compounds (e.g., hexanal) provide different aromas at low/high concentrations; (6) some compounds, when added together, provide a different aroma compared to when they are in single dilutions due to complex interactions in perception (synergic/masking/different aroma), and thus the resulting aroma cannot be reduced to the sum of the partial aromas given by each volatile compound; (7) there is a strong dependence on the individual when defining an aroma (e.g., green or rancid for hexanal and nonanal) in the perception of some aromas and the RM of aroma should be perceived as homogeneously as possible by all assessors smelling it.

Although the strategy described herein was designed for winey-vinegary and rancid aroma, it is valid to continue developing RMs for the other sensory defects of VOOs, which also need RMs for panel training and control. Once the RMs are developed, the next step, as described in Figure 2, is their validation using an evaluation process by panelists, ideally from panels from different parts of the world. This last step is currently being carried out by OLEUM project. Although the use of RMs is already described in current IOC norms for organoleptic assessment, it would be desirable to establish a perfected procedure for implementation of the RMs. Finally, the use of artificial RMs described in this work has the advantage of high reproducibility and availability since it is suitable for a defined formulation that can be produced on demand and/or marketed. Their use does not exclude the use of the current natural RMs if they are available to combine training in recognition of the defects, in the contextualization of a real oil and reproducible evaluation of their intensity.

## Figures and Tables

**Table 1:** Volatile markers of VOO sensory defects winey-vinegary and rancid, sensory characteristics and corresponding odor threshold relating to an oil matrix.

Sensory Defect	Volatile Marker	Sensory Characteristics	Odor Threshold (mg/kg)
Winey-vinegary	Acetic acid	Vinegary, sour	0.50
	Ethyl acetate	Sticky	0.94
	Ethanol	Alcohol	30.00
	3-Methyl-1-butanol	Whiskey	0.10
Rancid	Pentanal	Oily	0.24
	Hexanal	Oily, fatty	0.08
	Heptanal	Oily, fatty	0.50
	*E*-2-Heptenal	Tallowy, oxidized	0.04
	Octanal	Fatty	0.32
	Nonanal	Waxy, fatty	0.15
	*E*-2-Decenal	Fishy, fatty	0.01
	Hexanoic acid	Rancid	0.70

**Table 2:** Relative areas of volatile compounds selected to emulate winey-vinegary and rancid defects in VOOs in a set of 63 samples and in RMs provided by the IOC for each of the defects (RM IOC).

Volatile Compound	Category	N	Mean (Min–Max)	RM IOC
Acetic acid	EV	12	0.212 (0.037–1.115)	8.688
	V-L (excluding those with winey-vinegary defect)	44	0.678 (0.045–8.485)	
	V-L (winey-vinegary as main defect)	7	1.506 (0.436–2.389)	
Ethanol	EV	12	0.423 (0.111–1.030)	0.581
	V-L (excluding those with winey-vinegary defect)	44	2.770 (0.083–53.688) ^a^	
	V-L (winey-vinegary as main defect)	7	4.381 (0.033–19.039)	
Ethyl acetate	EV	12	0.150 (0.021–0.530)	19.620
	V-L (excluding those with winey-vinegary defect)	44	0.345 (0.013–1.519)	
	V-L (winey-vinegary as main defect)	7	0.874 (0.236–3.149)	
Hexanal	EV	12	0.731 (0.116–1.497)	8.396
	V-L (excluding those with rancid defect)	31	0.680 (0.069–6.533)	
	V-L (rancid as main defect)	20	1.013 (0.065–3.064) ^b^	
Nonanal	EV	12	0.002 (tr–0.004) ^c^	0.074
	V-L (excluding those with rancid defect)	31	0.003 (0.001–0.009)	
	V-L (rancid as main defect)	20	0.009 (0.001–0.050)	

^a^ With outlier 28.140 (0.083–1119.060). ^b^ With outlier 9.178 (0.065–164.316). ^c^ tr, traces.

**Table 3:** Main formulations (volatile compounds and concentrations in mg/kg) emulating winey-vinegary aroma in VOO that resulted from modified simplex procedure and were evaluated by assessors in terms of suitability as possible RM. Note: **1**, ethyl acetate; **2**, ethanol; **3**, acetic acid; **4**, hexanal; **5**, 1-hexanol; **6**, *E*-2-hexenal; **7**, *E*-3-hexen-1-ol.

1	2	3	4	5	6	7	Sensory Descriptors	Code
80	600	40	-	20	-	40	Slightly vinegary, sweet, slightly rancid	AV1
200	600	600	50	50	50	50	Vinegary, glue, chemicals, wood	AV2
400	1200	1200	100	100	100	100	Vinegary, chemicals, glue, rancid, mushrooms	AV3
200	600	600	10	10	10	10	Vinegary, glue, no natural, slightly vinegary	AV4
40	5000	600	10	10	10	10	Slightly vinegary, vinegary	AV5
-	5000	600	1.5	1.5	1.5	1.5	Less intense to vinegary	AV6
-	10,000	1200	3	3	3	3	Vinegary at high and medium-high intensities	AV7
-	20,000	2000	5	5	5	5	Vinegary, alcohol, artificial vinegary	AV8
-	32,000	4800	7.5	7.5	7.5	7.5	Vinegary, high intensity of acetic acid and alcohol	AV9

**Table 4:** Main formulations (volatile compounds and concentrations in mg/kg) emulating rancid aroma in VOO that resulted from Modified Simplex Procedure and evaluated by a set of assessors about their eligibility as possible RM. Note: **1**, hexanal; **2**, nonanal; **3**, 1-hexenol; **4**, *E*-3-hexen-1-ol; **5**, *E*-2-hexenal; **6**, *E*,*E*-2,4-hexadienal; **7**, *E*,*E*-2,4-decadienal; **8**, *E*-2-decenal; * pentanoic acid (500 mg/kg) and hexanoic acid (500 mg/kg) were added to the formulation.

1	2	3	4	5	6	7	8	Sensory Descriptors	Code
600	80	20	40	-	-	-	-	Green aroma ending with slight rancid sensory notes	R1
100	-	100	100	100	600	600	600	Rancid but slightly green as well, a little sweet, undesirable	R2
50	-	50	50	50	600	600	600	Rancid but slightly green, rancid but a little sweet	R3
35	-	35	35	35	600	600	600	Slight green, slightly rancid, sweet, ripe fruity, rancid nuts, rancid aftertaste	R4
600	-	1.5	1.5	1.5	600	600	600	Intensely rancid, nuts	R5 *
600	-	1.5	1.5	1.5	-	363	420	Intensely rancid, nuts, seeds	R6 *
